# Bovine Oviduct Epithelial Cell-Derived Culture Media and Exosomes Improve Mitochondrial Health by Restoring Metabolic Flux during Pre-Implantation Development

**DOI:** 10.3390/ijms21207589

**Published:** 2020-10-14

**Authors:** Tabinda Sidrat, Abdul Aziz Khan, Myeon-Don Joo, Yiran Wei, Kyeong-Lim Lee, Lianguang Xu, Il-Keun Kong

**Affiliations:** 1 Department of Animal Science, Division of Applied Life Science (BK21 Four), Gyeongsang National University, Jinju 52828, Korea; tabindasidrat06@gmail.com (T.S.); jmd1441@gmail.com (M.-D.J.); weiyiran1230@gmail.com (Y.W.); xulianguang428@gmail.com (L.X.); 2 Center for Discovery and Innovation, Hackensack University Medical Center, Nutley, NJ 07110, USA; azizkhanuop@gmail.com; 3 The King Kong Corp. Ltd., Gyeongsang National University, Jinju 52828, Korea; 0920-0728@hanmail.net; 4 Institute of Agriculture and Life Science, Gyeongsang National University, Jinju 52828, Korea

**Keywords:** oviduct epithelial cells, exosomes, TCA-cycle, metabolism, bovine embryo

## Abstract

Oviduct flushing is enriched by a wide variety of nutrients that guide the 3–4 days journey of pre-implantation embryo through the oviduct as it develops into a competent blastocyst (BL). However, little is known about the specific requirement and role of these nutrients that orchestrate the early stages of embryonic development. In this study, we aimed to characterize the effect of in vitro-derived bovine oviduct epithelial cell (BOECs) secretion that mimics the in vivo oviduct micro-fluid like environment, which allows successful embryonic development. In this study, the addition of an in vitro derived BOECs-condition media (CM) and its isolated exosomes (Exo) significantly enhances the quality and development of BL, while the hatching ability of BLs was found to be high (48.8%) in the BOECs-Exo supplemented group. Surprisingly, BOECs-Exo have a dynamic effect on modulating the embryonic metabolism by restoring the pyruvate flux into TCA-cycle. Our analysis reveals that Exo treatment significantly upregulates the pyruvate dehydrogenase (PDH) and glutamate dehydrogenase (GLUD1) expression, required for metabolic fine-tuning of the TCA-cycle in the developing embryos. Exo treatment increases the influx into TCA-cycle by strongly suppressing the PDH and GLUD1 upstream inhibitors, i.e., PDK4 and SIRT4. Improvement of TCA-cycle function was further accompanied by higher metabolic activity of mitochondria in BOECs-CM and Exo in vitro embryos. Our study uncovered, for the first time, the possible mechanism of BOECs-derived secretion in re-establishing the TCA-cycle flux by the utilization of available nutrients and highlighted the importance of pyruvate in supporting bovine in vitro embryonic development.

## 1. Introduction

The oviduct fluid microenvironment is composed of plethora of growth stimulatory factors, immunomodulatory components and extracellular-vesicles/exosomes (Exo), which all are known to play an important role during a series of crucial reproductive events [1,2,3]. The oviduct epithelium contains the population of several ciliated and secretory cells that makes the oviduct fluid, which influences the whole journey from embryonic development into a successful adult [1]. Co-culture of bovine oviduct-epithelial cells (BOECs) with embryos and its extracted secretome in an in vitro system creates an environment conducive to fertilization and supports the early embryonic stages [4,5,6,7]. The use of a co-culture system of BOECs and its derivatives has been employed to overcome the developmental cessation of in vitro produced mammalian embryos, which ultimately improves the pregnancy rate and outcome of embryo transfer [4,8]. Loss of normal metabolic function is one of the causes of development cessation during in vitro produced (IVP) mammalian embryos [9,10]. The absence of maternal signaling cues in the in vitro medium renders the embryo unable to absorb the available nutrients from the external environment to overcome the increasing demand of ATP required for expansion and proliferation [11,12]. Thus, studies regarding the influence of oviduct secretions on mammalian embryo development to increase the quality and survivability must continue to bring about an improvement in IVP, as well as refinement of ART procedures.

The broad applications of BOECs-culture system and its derivatives considered as an important step towards the advancement of assisted reproductive technologies (ART). To date, efforts have been made in the formulation of culturing media for the successful in vitro development of pre-implantation embryos [13]. Although the conventional in vitro microenvironment is formulated to closely mimic the in vivo oviductal fluid composition, the currently available media still lack various unknown oviductal fluid proteins and exosomes (Exo) [14]. Exo are very important bioactive molecules present in the oviduct fluid, generated from the secretion of epithelial cells. These nano-sized shuttles are able to cross the embryonic membrane and play an important role in modulating the pre-implantation embryonic development, as well as in establishing cross-talk between embryo–maternal interactions [11,14,15,16]. Exo are encapsulated by phospholipid bilayer membrane and are loaded with proteins, lipids, pool of RNA species (mRNA, miRNA, long noncoding RNA), and DNA fragments. These regulatory bioactive molecules enable the exosomes to mediate the responses of several signaling pathways associated with physiological and pathophysiological functions among neighboring cells and distant cells via extracellular environment [5,11,12]. Absorption of Exo by embryos from the extracellular environment provides a new insight into the intracellular communication between the oviduct epithelium and the developing embryo through an exogenous biomolecule delivering tool.

The developing embryo undergoes several important morphological and transcriptional event changes during its journey through the oviduct [1,17,18]. Several studies showed the positive influence of using BOECs and their secretome on early embryonic development; these can be observed in terms of developmental competence, cryotolerance, timing of embryonic genome activation, acquisition of epigenetic modifications, and cellular proliferation [12,16]. It has been demonstrated that during in vitro culture of mammalian embryos, especially in ruminants, the development arrested at the morula stage of 8–16 cell compaction [19], whereas the use of co-culture system of oviduct epithelial cell or its secreted condition medium overcome this developmental block and increases the likelihood of survival of IVP embryos [18]. This evidence shows that BOECs’ secreted factors have a remarkable effect, changing the nutrients and metabolite of the culture media through an exchange of signals between the developing embryo and the external environment [18]. However, a number of compelling pieces of evidence have shown the advantageous effects of BOECs-derived condition medium (CM) and BOECs-isolated Exo on the qualitative yield of IVP embryos [5,12,14], but the molecular mechanism involved in the oviduct–embryo dialogue to impact the metabolic responses of the developing embryos remains unknown.

Considering the metabolic response of the developing embryos, a study on the in vitro development of mouse embryos showed a preference for pyruvate uptake during initial pre-implantation stages, although the glucose is also essential during the compacted morula to BL transition [20]. After compaction, the glucose uptake increases, whereas the requirement of pyruvate uptake also remained high to support development beyond morula stage [10,20]. Studies on human in vitro embryonic development also suggested the need for pyruvate throughout development to the BL stage [21]. These highlighted that the growth medium has a significant influence on energy metabolism during preimplantation development, which improves mitochondrial health and the developmental competency of in vitro produced embryos [10,22]. Pyruvate is an essential molecule of the mitochondrial TCA (tricarboxylic acid) cycle for the generation of ATP [23]. Blocking the entry of pyruvate into TCA-cycle disturbs the metabolic flux and causes mitochondrial dysfunction [9,22]. Development arrest at the compaction stage in vitro might be the result of insufficient pyruvate for the TCA cycle.

The in vivo microenvironment of oviduct fluid provides several important biological molecules—lipids, proteins, and essential amino acids—to support normal metabolic flux throughout embryonic development [3,4]. However, the current evidence on oviduct secretions and intercellular communication between embryo and oviduct still requires conclusive studies. The current study focuses on the use of BOECs-monolayer derived CM and Exo, with a special emphasis on their role in improving the metabolic flux during in vitro embryonic development. Our study shows that the addition of BOECs-CM and BOECs-Exo to the in vitro culture medium restores the pyruvate flux by blocking its upstream inhibitor and upregulate the pyruvate dehydrogenase (PDH) and glutamate dehydrogenase 1 (GLUD1) expression. Our study, for the first time, provided an explanation for the molecular mechanism of IVP embryos cultured in the presence of BOECs-CM and Exo, which greatly influence the metabolic response of embryos by re-establishing the pyruvate flux that ultimately improves the mitochondrial functioning. Our study establishes an important link between energy metabolism and morula arrest by using BOECs-monolayer derived CM and Exo that dislodges the development block of the compacted morula stage.

## 2. Results

### 2.1. Maintenance of BOEC Monolayer Typical Epithelial Cell Morphology and Characteristics

**Figure 1:**
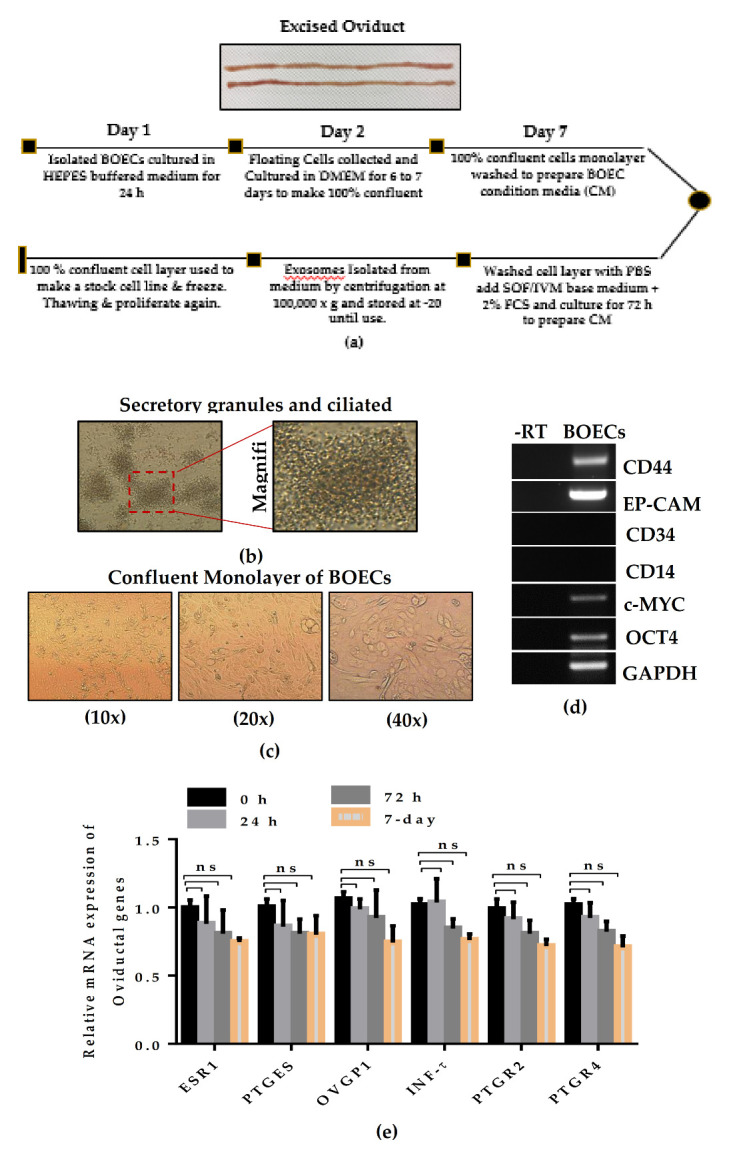
Morphology and characterization of bovine oviduct epithelial cells (BOECs). (**a**) Schematic flow diagram of BOECs isolation and culture. (**b**) Aggregated ciliated cells after 24 h culture, with the intense appearance of beating cilia like structure on the cell surface. (**c**) Isolated BOECs-monolayer showing typical epithelial morphology after several days of culture. (**d**) RT-PCR displaying the presence of characteristics epithelial and cell proliferative marker and the absence of hematopoietic (CD34 and CD14) markers from cell culture. (**e**) qRT-PCR analysis of the expression profile of several oviduct marker genes in cultured BOEC monolayer at 0, 24, 72 h and seven days of cell seeding. Data are presented as a result of ± SEM (standard error of mean) of four replicates. ns, indicates non-significant difference.

To mimic the in vivo-like microfluidic environment during in vitro production of embryos, we first isolated and stably cultured the BOECs in a defined condition medium for 7 days until they formed a tightly confluent monolayer (Figure 1a). The oviduct epithelium consists of an abundant population of ciliated and secretory cells. In resemblance to natural characteristic features, the results of light microscopy showed cell aggregates during the first 24 h culture of BOECs, with typical morphology of secretory and ciliated cell population detected by the presence of vigorously beating cilia on the apical surface of all cell aggregates (Figure 1b). Epithelia polarization is further characterized for 6–7 days culture of BOECs that allow the cells to grow coherently for 6–7 days culture period and finally settle as a tight, confluent monolayer sheet (Figure 1c). The cultured BOECs were further characterized by the presence of several markers for the determination of epithelial lineage, cell culture purity, and proliferation potential. The strong expression of CD44 and EP-CAM highlighted the cells are of epithelial origin. Whereas the non-detectable expression of CD34 and CD14 showed the absence of hematopoietic progenitor cells in the isolated BOECs culture population. Also, the presence of c-MYC and OCT4 explained how the cells maintain their proliferation potential throughout the 6–7 day culture period (Figure 1d). Moreover, the functional morphology of BOECs-monolayer was further validated by the expression of oviduct-specific genes makers. The results show that the BOECs-monolayer maintained the characteristics oviduct genes marker throughput the culture period; although the expression was slightly downregulated, but there was no statistical difference during long term culture (Figure 1e). All these results provide evidence of the typical physiological feature of BOEC monolayer and suggest the appropriate maintenance of oviduct cell culture system during in vitro processes.

### 2.2. Oviductal Secretion are Enriched in Oviductosomes/Exosomes

**Figure 2:**
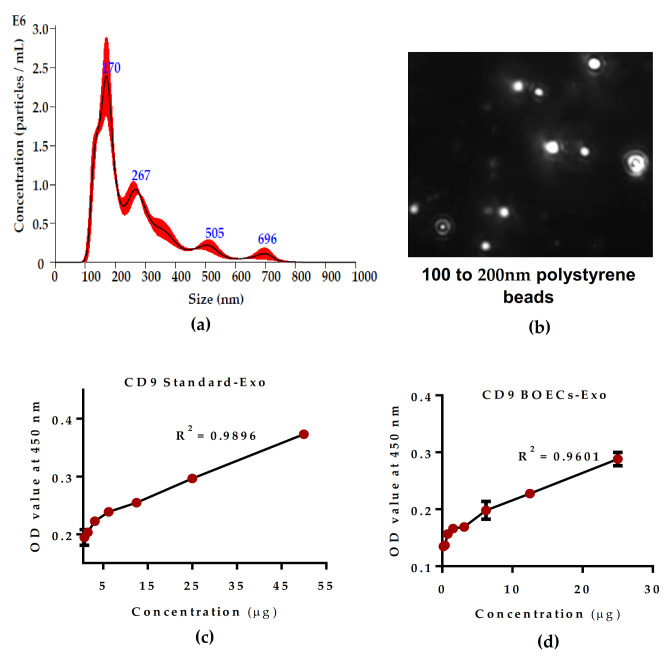
Characterization and quantification of BOECs-derived exosomes (Exo). (**a**) Size distribution of BOECs-derived Exo measured by nanoparticle tracking analysis (NTA) using 100× sample dilution. Graphical representation of data showing the quadratic interpolation of mean number of particles present in 100× dilution sample. (**b**) Screen shot of NTA of 100–200 nm beads. (**c**,**d**) ELISA graph presenting the quantification of BOECs-derived Exo protein concentration. OD values of Exo-specific CD9 antibody represents the concentration of protein. The Standard-Exo used to generate the calibration curve for quantitation of Exo carrying CD9 from Exo-ELISA data. Data are presented as a result of ± SEM (standard error of mean) of three replicates.

Next, we wanted to determine the effect of oviduct epithelial cells’ secretion on early embryonic development. In vivo, the oviduct luminal fluid is enriched in a wide variety of secretory factors that influence the early embryonic development. To exclusively determine the effect of Exo during pre-implantation development, we performed a biophysical and molecular characterization of Exo obtained from the confluent in vitro cultured BOECs monolayer (Figure 2). Nanoparticle tracking analysis (NTA) was performed to reveal the size and concentration of BOECs-monolayer derived Exo. The analysis revealed that Exo range 80–150 nm in size and have an average concentration of 3 × 10^8^ particles per mL (Figure 2a). The size and distribution of particles were also presented in terms of intensity and visualized as shown in the screenshot captured by NanoSight LM10 (Figure 2b). In addition, the BOECs monolayer-derived Exo enrichment was further verified and quantified with CD9 Exo-specific antibody through Exo-ELISA colorimetric assay (Figure 2c,d). All together, these results suggest that an in vitro cultured BOECs monolayer could be maintained well in a functional state and that its secretion is capable of generating abundant Exo.

### 2.3. BOECs-Derived CM and Exosomes Reduces the Embryonic-Development Block and Enhances the In Vitro Produced BLs Quality and Yield

**Figure 3:**
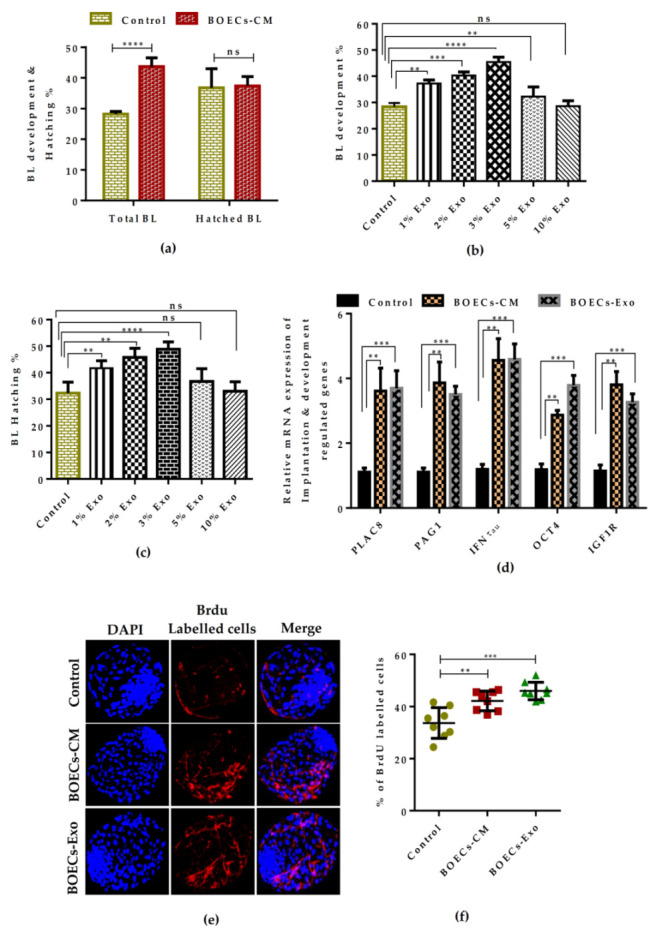
Effect of BOECs monolayer derived CM and Exo supplementation on bovine BL yield and quality. (**a**,**b**) Representative bar graph showing the development and hatching % of BLs cultured in the absence or presence of BOECs-CM and different concentration of Exo. Data are presented as a result of ± SEM (standard error of mean) of 11 and 8 replicates, respectively. (**c**) qRT-PCR analysis of the expression profile of several BL implantation potential and development competence-related genes in response to BOECs-CM and 3% Exo supplementation. (**d**,**e**) Immunofluorescence analysis showing the labelling of 5-bromo-2′-deoxyuridine (BrdU) in day-8 BLs cultured in the absence or presence of BOECs-CM and 3% Exo. (**f**) Quantification of BrdU labelled cells. Bar graph data represent means ± SEM (standard error of mean) from triplicate experiments, performed with *n* = 20 BLs per group in each replicate. ** p < 0.01; *** p < 0.001; **** p < 0.0001 denotes significant difference. Original magnification is 100×.

To better understand that how oviduct secretions influence the early embryonic development, we determined the practical application of condition media as well as the oviductosomes/exosomes generated from in vitro-cultured BOECs monolayer during bovine pre-implantation development. Our supplementation resulted in a noticeable improvement in BLs yield and hatching ability (BOECs-CM: 43.6 ± 0.86; 37.5 ± 0.85, respectively) as shown in Table 1 and Figure 3a. The supplementation of BOECs derived Exo showed a dose-dependent impact on embryonic development; for instance, the addition of 3% Exo during maturation and embryo culture significantly improved the embryo quality in terms of BLs formation rate and hatching ability (BOECs-Exo: 45.4 ± 0.68; 48.9 ± 0.97, respectively) as shown in Table 2 and Figure 3b,c. Also, the development of 8–16 cell stage embryos were remarkably enhanced with the supplementation of both BOECs-CM and Exo as shown in Tables S1 and S2. To determine the effect of in vitro derived BOECs secretions on development and implantation regulated genes expression, we performed q-RT-PCR analysis of BLs cultured in the presence of BOECs-CM and Exo. The results showed that BOEC’s secretory milieu remarkably enhances the various development- and implantation potential-related genes expression in embryos produced in BOECs-CM and Exo supplemented medium compared to control embryos (Figure 3d). To further evaluate the quality of embryos, we determined the total cell abundance. Our analysis indicated that both BOECs-CM and Exo-derived embryos have significantly higher cell proliferation ratios than embryos developed in control culture medium (Figure 3e,f). All these results indicate that CM and Exo from in vitro cultured BOECs mimics the in vivo oviduct flushing and are capable of beneficially influencing the in vitro culture conditions for production of bovine embryos.

### 2.4. Co-Culture of Embryos with BOECs-CM and Exosomes Reduces the Cellular Stress and Improves the Cell Survival Ratio

**Figure 4:**
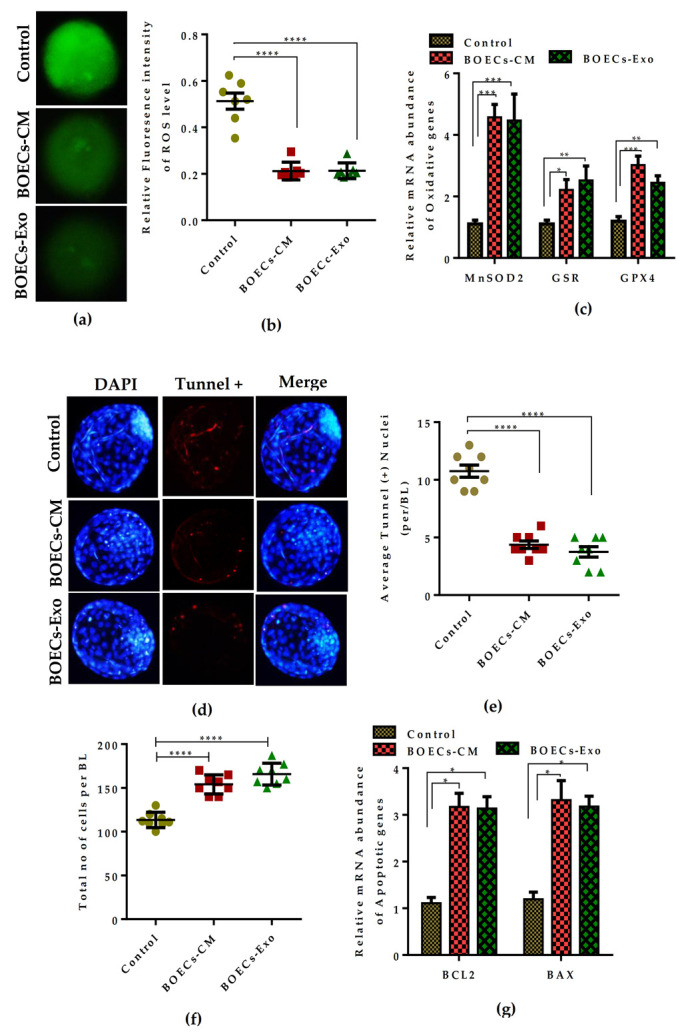
Effect of BOECs-CM and Exo supplementation on generation of ROS level and cell apoptotic index during bovine BL development. (**a**) DCHDFA staining showing the accumulation of ROS in day-8 BLs cultured in the presence of BOECs-CM and 3% Exo. (**b**) Bar graph data represents the quantification of fluorescence intensities. (**c**) Relative mRNA level of MnSOD1, GLUT1, and G6PD in control, BOECs-CM, and 3% Exo cultured groups. (**d**) Fluorescence images showing the TUNEL-positive cells (red) and DAPI (blue) in control, BOEC-CM, and 3% Exo supplemented groups. Apoptotic cells in nuclei are indicated with white arrows. (**e**,**f**) Bar graph data represents the average TUNEL (+) nuclei per BLs and total cell count in the indicated groups. (**g**) qRT-PCR analysis showing the expression of representative apoptotic genes in the indicated groups. Data presented as means ± SEM from triplicate experiments with *(n* = 15 BLs) per group in individual set of ROS and TUNEL assays. For mRNA expression analysis *n* = 5 BLs/group used in triplicate experiments. * p < 0.05; ** p < 0.01; *** p < 0.001; **** p < 0.0001 indicates significant difference. Original magnification of representative images is 100×.

To determine the impact of in vitro-derived BOECs-CM and Exo on cellular oxidative stress and damage during pre-implantation development of bovine embryos, we measured the production of reactive oxygen species (ROS) level. Bovine embryos with the addition of BOECs-CM and Exo remarkably encountered the subsequent rise in the ROS level during the in vitro culture period compared to the control cultured medium embryos (Figure 4a). Quantification of ROS florescence intensity showed in Figure 4b. Concomitantly, we examined the mRNA abundance of well-known antioxidant enzymes. The analysis indicated that embryos cultured in the presence of BOECs-CM and Exo have significantly higher expression of antioxidant transcripts relative to the embryos cultured without BOECs-CM and Exo supplementation (Figure 4c). Next, we performed the respective apoptotic assay to test the DNA damage level. Results indicated more TUNEL-positive nuclei in the control embryos developed in conventional cultured media, while BOEC-CM and Exo supplementation led to significantly fewer apoptotic cells observed during fluorescence imaging (Figure 4d). Quantification of average TUNEL-positive nuclei versus the total cell number, with significant differences among the groups are presented in Figure 4e,f. Moreover, the effect on cell death and damage was further confirmed by the expression of anti-apoptotic marker genes. These results suggested that higher mRNA expression of BCL2 and BAX in the BOEC-CM and Exo supplemented groups inhibited the induction of apoptosis in embryos compared to the control group (Figure 4g). All together, these results provided evidence that BOEC secretions contain some additional factors that have a protective role and enhance the cell survival ratio in early developing bovine embryos during extended periods of in vitro culture.

### 2.5. BOECs-Derived CM and Exosomes Re-Establish the Pyruvate Flux and Improves the In Vitro-Produced Embryo Metabolism

**Figure 5:**
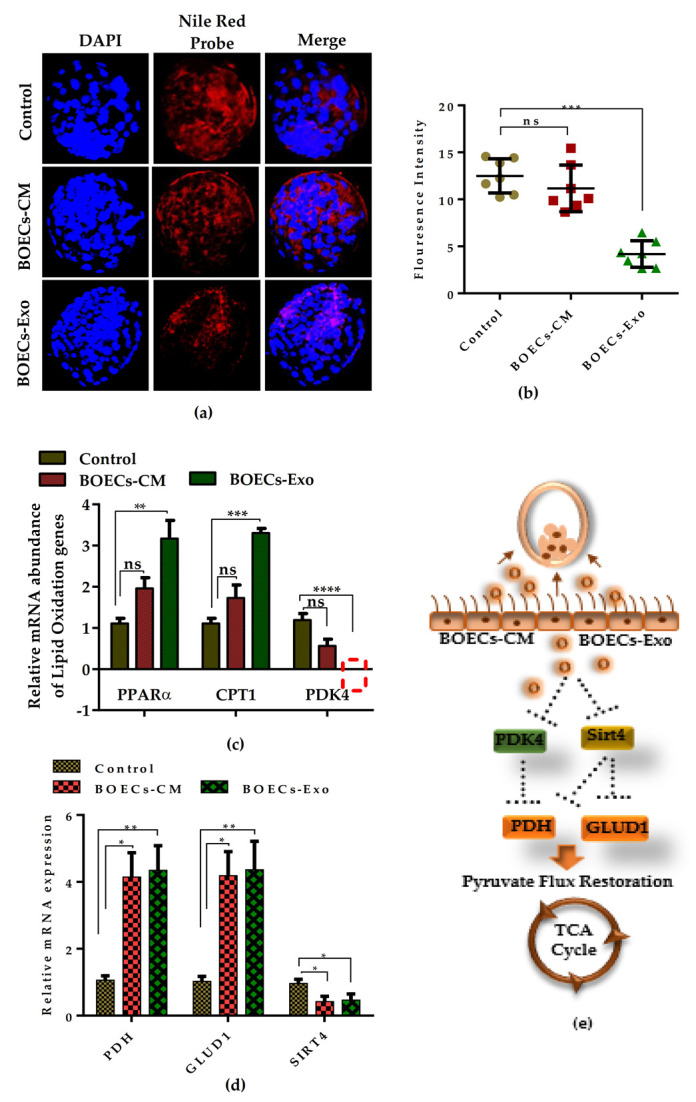
Effect of BOECs-CM and Exo supplementation on utilization of FAO and TCA cycle metabolism during in vitro culture of bovine embryos. (**a**) Fluorescent Nile probe (red) staining.displaying the accumulation of lipid content in day-8 BLs in control, BOECs-CM and Exo supplemented groups. (**b**) Quantification of fluorescence intensities presented as means ± SEM in each group (*n* = 20). Original magnification 100×. (**c**,**d**) The qRT-PCR analysis presenting the relative mRNA expression of lipid and TCA cycle-metabolizing genes in day-8 BLs cultured in indicated condition. Data in the bar graphs presented as means ± SEM from three independent sets of experiments including (*n* = 5) BLs per group in each replicate. * p < 0.05; ** p < 0.01; *** p < 0.001; **** p < 0.0001 denotes significant difference among the groups. (**e**) Hypothetical model illustrates the mechanism of action of BOECs-CM and Exo supplementation effect on developing embryo by utilization of lipids and glucose metabolism. The addition of CM and Exo restore the pyruvate flux into the TCA cycle by blocking its upstream inhibitors.

To analyze the effect of BOECs-CM and Exo on the utilization of fatty acid oxidation (FAO) metabolism, we first determined the lipid content of the in vitro-cultured embryos from the control as well as BOECs-CM and Exo supplemented group. Confocal imaging by a lipid specific florescent probe showed that BOECs-CM supplemented BLs have same lipid content as the control BLs whereas Exo supplementation significantly oxidizes the lipid droplet in developing embryos (Figure 5a). The florescent intensities are shown in Figure 5b. To confirm this analysis, we assessed the expression of several lipid-metabolizing genes. The qRT-PCR analysis revealed that expression of PPARα, CPT1, and PDK4 in control and BOECs-CM cultured embryos were not statistically different. Interestingly, Exo supplementation led to a significant increase in CPT1 expression and an inhibitory effect on PDK4 expression (Figure 5c). PDK4 is the key regulatory enzyme of glucose and fatty acid metabolism that regulates the entry of pyruvate into tricarboxylic acid (TCA) cycle [24]. Furthermore, we performed the analysis to determine any changes in the expression of TCA-cycle-metabolizing genes. The analysis revealed that BOECs-CM and Exo supplementation significantly enhances the pyruvate dehydrogenase (PDH) and glutamate dehydrogenase 1 (GLUD1) expression, whereas it downregulates the expression of Sirt4 gene, and a significant effect was observed in the Exo-exclusive supplemented group (Figure 5d). In a schematic model, it is suggested that the suppression of Sirt4 and PDK4 expression in the pre-implantation embryos cultured with the addition of BOECs-CM and Exo might improve the entry of pyruvate into TCA cycle by up regulating the expression of TCA metabolizing enzymes. Both PDH and GLUD1 fuel the TCA cycle which ultimately provides enough energy to support the pre-implantation stages of the developing embryo (Figure 5e).

### 2.6. BOECs-CM and Exosome-Mediated Metabolic Flux Improves Mitochondrial Functioning during Embryonic Development

**Figure 6:**
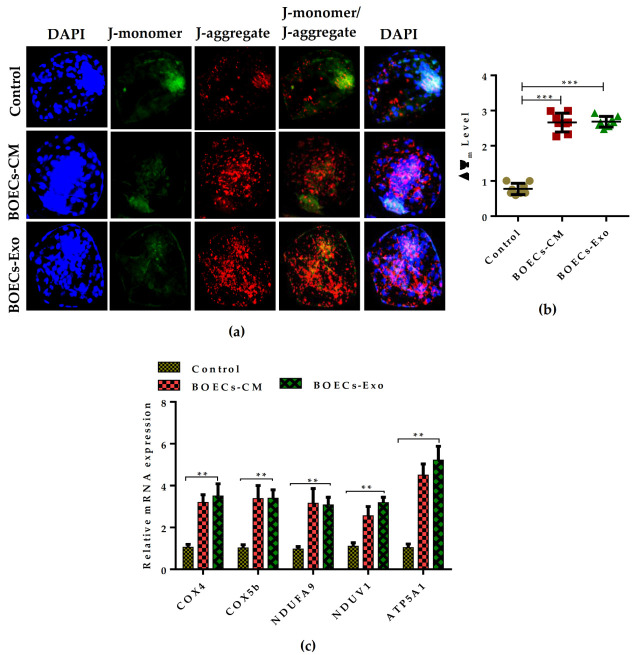
Supplementation of BOECs-CM and Exo enhances the mitochondrial functions during bovine embryonic development. (**a**) Fluorescence confocal microscopy images of the JC-1 staining, showing the mitochondrial ∆Ψ_m_ detected by the presence of J-monomer (green) and J- aggregates (red) in control, BOECs-CM, and Exo groups. Nuclei stained in blue. Original magnification 100×. (**b**) Quantification for relative fluorescence intensities were presented in graph. BLs (*n* = 20) analyzed per group in triplicate sets of each experiment. (**c**) Relative mRNA expression profile of mitochondrial OXPHOS subunit and ATP synthesizing genes analyzed in day-8 BLs cultured in the absence or presence of BOECs-CM and 3% Exo. ** p < 0.01; *** p < 0.001 represents significant difference.

Based on our results, which suggested the improvement of TCA cycle metabolism in developing embryos cultured in the presence of BOECs-CM and Exo supplementation, we aimed to determine the mitochondrial function by analyzing the mitochondrial membrane potential (∆Ψ_m_). The results indicated a remarkable difference in the mitochondrial activity in the control embryo group versus BOECs-CM and Exo supplemented groups, which showed the presence of higher J-aggregate ratio (Figure 6a). Quantitative difference in the florescence intensities of mitochondrial ∆Ψ_m_ are visible in Figure 6b. Moreover, we checked the mitochondrial metabolic activity by analyzing several mitochondrial oxidative phosphorylation (OXPHOS) subunit gene expression and ATP-synthesizing marker. Similarly to the mitochondrial ∆Ψ_m_, the embryos cultured in the presence of BOECs-CM and Exo-supplemented media showed a significantly higher expression profile of several mito-OXPHOS subunit genes and increased mRNA transcript of ATP synthesizing enzyme relative to the control culture medium embryos (Figure 6c). All these results suggested that BOECs-CM and Exo greatly impact the in vitro cultured condition and significantly improve the developing embryo quality by helping it to establish its own metabolism.

## 3. Discussion

In this study, we demonstrated the application of a co-culture system of BOECs monolayer generated CM and BEOCs-derived Exo to support the in vitro embryonic development. Our study revealed the systematic approach of in vitro culturing of BOECs and the use of its derivative secretions, specifically BOECs-derived Exo, not only improve the standard IVP conditions but also help to improve our understanding of how, in vivo, the concentration of soluble factors influences the early embryonic developmental processes.

Previously, it has been shown that BOECs can be maintained in a functional state in the absence of serum; however, it showed some morphological degenerative signatures such as loss of secretory granules in FCS-free medium [4]. To avoid the significant loss of characteristic secretory features, we cultured the BOECs in a complete growth standard culture medium according to the composition previously described by [17]. The BOECs sheets were well maintained for up to seven days’ culture period and analysis showed the existence of full functional biological activity with typical epithelial characteristic morphology (Figure 1). The detection of ciliated cells shows the maintenance of cell polarity, fundamental for the architecture and function of epithelial cells [4]. It was documented that naturally neutral lipids were present in the bovine oviduct epithelium that were used as a source of energy for their growth and proper functioning [13]. Thus, the addition of serum not only allows the better attachment of the proliferating cells, but has been proven to be required during the in vitro culture of BOECs [25]. On the other hand, a high concentration of serum was associated with a low quality of embryo production, higher incidence of apoptosis that leads to early embryonic death or either survived fetus produced with several developmental anomalies [18,26]. Thus, in order to minimize the effect of serum on embryo quality, we reduced the concentration of FCS (2%) for the production of CM enough to support the growth of a well-settled 100% confluent BOEC monolayer for 48 h. The results showed that BOECs-CM not only remarkably improve the BL formation rate but also did not impair the quality of the BLs, such as the hatching ability relative to the control (Table 1 and Figure 3a). These results are in agreement with a previous investigation that reported that a low concentration of FCS (2.5%) did not interfere with the survival and conception rate of BLs after cryopreservation and embryo transfer, respectively [27].

It is well known that, from compaction to the BL stage, nutrients in the oviduct fluid play a role in the transit of embryonic metabolism via maintaining the oviduct-embryo dialogue [1]. The oviductosomes/exosomes are the main cargo present in the oviduct fluid that establishes the embryo–maternal-crosstalk, which results in better utilization of nutrients from the luminal fluid and help the embryos to establish their own metabolism [11,14]. Our results from the BOECs-Exo exclusive supplementation group showed a significant beneficial effect on early embryonic development, dose dependently, whereas the excessive concentration of Exo above 3% impaired the development and hatching rate of BLs (Table 2 and Figure 3b,c). The presence of an excess Exo concentration in the culture media might lead to a perturbed metabolic pathway during the pre-implantation period of embryonic development. The regulation of metabolism plays a central role when considering the interaction of embryo with its environment [10]. Interestingly, the supplementation of in vitro derived BOECs-Exo had a remarkable effect on metabolism-related genes expression during in vitro cultured bovine embryos. During embryonic development within the oviduct, the nutrient composition of the oviduct fluid protect the developing embryo from the oxidative stress, either by secreting free radical scavenging proteins, or by modulating antioxidant enzymes’ level to provide protection against the oxidative damage [28,29]. The perturbed redox balance and insufficient antioxidants levels are the major cause of DNA fragmentation which ultimately accelerates cell death and results in poor quality of embryos [29]. The production of ROS level, the expression of several oxidative metabolism genes, and the cell apoptotic ratio were remarkably reduced with the addition of BOECs-CM and Exo (Figure 4a–c), which reflects that developing embryo might have shifted the utilization of energy from oxidative to glycolytic metabolism due to the availability of nutrients in the culture media.

During oocyte maturation and development of mammalian embryo, substantially, oxidation of fatty acids provide higher amount of energy in the form of ATP by converting it into acetyl-CoA [30,31]. The in vitro cultured BLs supplemented either with BOECs-CM and Exo were analyzed for lipid droplet accumulation and FAO metabolizing genes’ expression; both groups showed similar expression pattern, but the effect was significantly more pronounced in the BOECs-Exo supplemented group (Figure 5). The addition of serum during preparation of BOECs-CM may explain the reason for the presence of accumulated lipid droplets in BOECs-CM derived embryos. The major FAO metabolism regulating genes, such as PPARα and CPT1, are highly upregulated in Exo-treated BLs. The statistically insignificant difference between BOECs-CM and the control may reflect the effect of serum in the culture media. This also explains that how BOECs-derived secretions regulate the FAO metabolism in the developing embryo by promoting PPARα and CPT1 expression. PPARα is found to be the upstream regulator of CPT1 and stimulates its transcription by binding with PPAR responsive element on the promoter region of CPT1 gene [32], thus the analysis showed a concomitant increase in the mRNA expression of both genes. The key regulatory enzyme of the metabolic pathway is PDK4, which is known to play an important role in maintaining metabolic flux by regulating the amount of pyruvate entering into the TCA cycle through the inhibition of pyruvate dehydrogenase (PDH) [24]. PDH is the main enzyme that regulates the conversion of pyruvate into acetyl-CoA and leads to the synthesis of a higher amount of ATP to meet the metabolic demands of the developing mammalian embryo [9,23]. Surprisingly, supplementation of BOECs-derived Exo significantly enhances the expression of PDH by blocking its upstream inhibitors PDK4 and SIRT4 (Figure 5c,d). SIRT4 serves as a key metabolic sensor that inhibits the PDH and GLUD1 activity [10]. Sirt4 is also an upstream inhibitor of PDH and GLUD1 [33,34]. The enhanced expression of PDH and GLUD1 suggests the removal of a PDK4 and SIRT4 inhibitory block, which ultimately restores the pyruvate flux to fuel the TCA cycle during embryonic pre-implantation development.

More frequently, it has been reported that development and quality of an embryo are directly co-related with its metabolism [10]. Our observations showed that the effect on the glycolytic metabolic pathway was more pronounced in the Exo exclusive treatment group than in the BOECs-CM supplementation group. These effects of Exo treatment echo a similar effect, the improvement of the TCA cycle which was reported in Exo derived from mesenchymal stromal cells, improved the mitochondrial function deficiencies in an in vitro culture model of human pulmonary artery smooth muscle cells [35]. The upregulation of TCA cycle metabolizing genes is a signature of improved mitochondrial health [9,10]. It is also well known that, in developing embryos, the transition from oxidative to glycolytic metabolism is accompanied by the maturation of mitochondrial function [22]. In our results, the mitochondrial metabolic activity was significantly improved in embryos cultured in a BOECs-CM and Exo supplemented medium. The embryos have remarkably higher expression of OXPHOS and ATP-synthesizing genes (Figure 6). These observations suggested that BOECs-derived-CM and Exo have a significant impact in terms of improving the mitochondrial heath of in vitro-produced bovine embryos.

In conclusion, our results suggest a possible mechanism in the in vitro cultured BOECs-derived-CM and Exo produced embryos in enhancing the metabolic flux. The expression of several TCA-cycle metabolizing genes were not the same as the genes expressing the flux of molecules involved in the metabolic pathways. These genes improve the metabolism and lead to better in vitro embryonic development. These findings highlight that the understanding of several embryologic nutrient requirements is necessary during the preparation of commercially synthesized media, which mimics the in vivo oviductal fluid composition and fully supports the early embryonic stages of development. Regarding the role of Exo, there are several possible mechanisms by which BOECs-Exo exerts a positive effect to influence the embryonic development. Exo effects on embryonic development varied according to the content of medium used for the primary culture of BOECs [14]. These observations suggested that a detailed proteomic analysis of in vitro as well as in vivo derived BOECs-Exo is needed to further decipher the role of Exo and its application during in vitro culture.

## 4. Materials and Methods

All chemicals/reagents used in assays were purchased from Sigma Aldrich (St. Louis, MO, USA) unless otherwise specified.

### 4.1. Isolation and Culture of Bovine Oviduct Epithelial Cells (BOECs)

Cow oviducts were collected from a local abattoir under the legislation of Institutional Animal Care and Use Committee of Gyeongsang National University (Approval ID: GAR-110502-X0017; date: 02-05-2011). Excised oviducts from slaughter house were immediately transported to the laboratory within two hours in ice cold DPBS (Dulbecco’s phosphate buffered saline). Oviducts were washed thrice in cold DPBS supplemented with 100 U/mL penicillin plus 100 μg/mL streptomycin and dissected free of surrounding tissues. BOECs were collected and cultured as described by [17]. In a brief BOECs were isolated in HEPES-buffered Medium 199 supplemented with 100 U/mL penicillin and 100 μg/mL streptomycin by scrapping and squeezing the oviductal contents out of the ampullary end of the oviducts. The retrieved cells were washed twice by centrifuging for 550× *g* at 25 °C for 5 min in HEPES-buffered Medium 199 containing 100 IU/mL penicillin and 100 μg/mL streptomycin. For 24 h the clean cells with minimal blood contamination were cultured in HEPES-buffered Medium 199 supplemented with 100 U/mL penicillin, 100 μg/mL streptomycin, and 10% fetal calf serum (FCS; Bovogen Biologicals, Melbourne, Australia, was centrifuged at 100,000× *g* at 4 °C for 60 min to deplete the extracellular vesicle content from FCS, aliquoted and stored at −20 °C for further use). Within these 24 h, the cells formed floating vesicles with actively beating cilia collected by centrifugation at 550× *g* at 25 °C for 5 min. The cells were suspended in DMEM/Ham’s F12 medium (DMEM/F12 Glutamax I, Gibco BRL, Paisley, UK) supplemented with 5 μg/mL insulin, 5 μg/mL transferrin, 10 ng/mL epidermal growth factor, 50 nM trans-retinoic acid, 10 mM glutathione, 100 μg/mL gentamycin, 5% FCS, and 2.5 mg/mL amphotericin B. The cells were mechanically separated with the aid of pipette. Cells were then cultured at final concentration of 3 × 10^6^ cells/mL in 6-well plates at 38.5 °C in an atmosphere of 5% CO_2_ with saturated humidity until confluence within 6–7 days. The 100% confluent monolayer was washed with PBS and used for the generation of condition media.

### 4.2. Isolation of Oviduct Extracellular Vesicles (Oviductosomes/Exosomes)

Conditioned media obtained from a 100% confluent BOEC-monolayer were pooled into a 50 mL conical tube. Exo were purified by sequential centrifugation of the conditioned media as described by [36], with little modification of the centrifugation length and speed. In brief, the filtered condition media was first centrifuged at 300× *g* for 10min, followed by 10,000× *g* for 30 min to remove dead cells, debris, and contaminating proteins. The pellet discarded and clean supernatant was ultra-centrifuged at 100,000× *g* at 4 °C for 60 min in a (BECKMAN L8-M; SW41T1 rotor, USA) to pellet the Exo. The pellets were suspended in ice cold PBS, aliquoted and stored at −20 °C for further analysis and supplementation into in vitro culture media.

### 4.3. Nanoparticle Tracking Analysis (NTA) 

The software used for capturing and integration of the data was NTA version 3.4 Build 3.4.003 (Malvern, UK). For the analysis of BOECs-monolayer derived Exo, the 100× sample dilution in PBS was used. The sample loaded into the cell chamber of an instrument that measured each sample at different random position throughout the cell. The measurement was recorded in triplicate cycles at each position. The pre-assessment parameters were adjusted to sensitivity 85, viscosity (water) 0.9 cP, frame rate of 25 frames per second (FPS) and total of 1498 frames per sample was examined, camera type (sCMOS) with manual shutter speed and gain adjustment, laser type green with 100 laser pulse duration, 25 °C temperature, and 7.0 pH. The specification of post-acquisition parameters was as follows: minimum brightness 22, maximum pixel area 1000, and minimum area 10 pixels. All quality control parameters such as temperature, conductivity, electrical field, and drift measurements were adjusted by applying instrument optimized setting. The mean, median, and mode which indicated the diameter and size of the particles as well as along with their distribution/concentration in terms of particles per mL of the sample, were calculated by excluding the less accessible readings from the data. The curve in the graph indicated the number of particles per particle size was obtained by using quadratic interpolation.

### 4.4. Exo-ELISA Colorimetric Assay for Exo-Protein Quantification

For quantitative and qualitative analysis of Exo-protein concentration, a double sandwich enzyme-linked immunoassay (ELISA) ExoQuant^TM^ colorimetric kit from (cat no. K1205-100; BioVision, Milpitas, CA, USA) was used according to the manufacturer’s instruction. The standard Exo and BOEC-monolayer derived Exo sample was serially diluted and added into the wells of ELISA strips, then incubated overnight at 37 °C under humid atmospheric condition. After washing, it was incubated with primary antibody anti-CD9α (diluted in 1× sample buffer) at 37 °C incubator for 2 h. After washing again, incubated with streptavidin-HRP conjugated secondary antibody at 37 °C for 1 h. The content was washed off and incubated with chromogenic substrate solution for 10 min at room temperature under dark conditions. The reaction was stopped by adding a stop solution and the OD value at 450 nm absorbance with an ELISA-reader was measured. The standard curve was obtained by plotting the average concentration values from different standard concentrations against the corresponding amount of Exo. The mean absorbance for standard Exo and sample Exo was calculated in duplicate sets. The results of OD values were interpreted by linear regression analysis.

### 4.5. In Vitro Embryo Production

Immature cumulus oocyte complexes (COCs) were retrieved from follicles (2–8 mm diameter) from the ovaries of Korean native Hanwoo cows, obtained from the local slaughterhouse, and placed in physiological saline (0.9% NaCl) at 37.5 °C. The TL-HEPES medium was used for the collection of oocytes, and afterwards submitted to in vitro maturation (IVM) and in vitro fertilization (IVF) as described previously by [37]. In a brief, the collected oocytes were cultured for 22–24 h in NUNC 4-well plates (Nunc, Roskilde, Denmark) containing IVM medium, composed of tissue culture media-199 (TCM-199) supplemented with 10% (v/v) fetal bovine serum (FBS), 1 µg/mL estradiol-17β, 10 µg/mL follicle-stimulating hormone, 0.6 mM cysteine, and 0.2 mM sodium pyruvate. After following IVM, the matured COCs were inseminated with 1 × 10^6^ sperms/mL of frozen-thawed semen straws from Hanwoo Bulls (KPN-1175, NongHyup, Agribusiness Group Inc, Republic of Korea). Approximately 18–20 h of post-insemination, the presumptive zygotes were cleared from the cumulus cells by repeated pipetting and completely denuded presumptive zygotes were cultured for up to 8 days in synthetic oviduct fluid (SOF) medium. To support the in vitro culture (IVC), SOF media supplemented with 44 μg/mL sodium pyruvate (C3H3NaO3), 14.6 μg/mL glutamine, 10 IU/mL penicillin, 0.1 mg/mL streptomycin, 3 mg/mL FBS, and 310 μg/mL glutathione were used for culturing of embryos [38]. The embryonic development proceeded to the BL stage under humidified atmospheric condition of 5% CO_2_ at 38.5 °C were recorded and used for comparative analysis.

### 4.6. Supplementation of BOEC-CM and Exo during Embryo Culture

Forty-eight hours before starting the culture of embryos, the cell culture media was replaced with in vitro maturation medium (IVM) and synthetic oviduct fluid medium (SOF) supplemented with essential amino acids with addition of 2% FCS. To minimize the effect of serum concentration on embryonic development minimal dose of serum was used. After 48 h, the condition media (CM) from three different monolayers were pooled out. Three harvested collections were combined in a conical tube and centrifuged at 300× *g* for 10 min to remove all cell debris. The CM was then filtered through a 0.22 μm nitrocellulose membrane, aliquoted, and stored at 4 °C refrigerator. The BOECs derived CM was pre-warmed in 38.5 °C incubator before oocyte maturation and culture of embryos. For the supplementation of (Exo) during in vitro development of embryos, the 100× dilution of Exo was tested between the ranges of 1 to 10%. The supplementation of 3% Exo was observed to support the maximal embryonal development. To perform the further experimental analysis 3% Exo concentration was used. At day 8, the number of embryos developed to the BLs stage in BOECs derived-CM and BOECS derived-Exo media were recorded. Afterwards, BLs were washed three times in 1× PBS and either used live or fixed in 4% paraformaldehyde for further experimental analysis. For gene expression pattern, washed BLs in nuclease free water were immediately snap-frozen in liquid nitrogen and stored at −80 °C in Eppendorf tubes.

### 4.7. RNA Extraction and Real-Time PCR

The total RNA from BLs (*n* = 5 per group) was isolated with RNA isolation kit (PicoPure, Arcturus, ThermoFisher, Foster City, CA, USA) and used to synthesized cDNA with iScript reverse transcriptase (BioRad). The qRT-PCR analysis was performed as described by [38]. In a brief, the relative mRNA abundance of all genes was analyzed by real-time quantitative (q)RT-PCR with SYBR Green master mix using Cycler BioRad system. Threshold (Ct) values normalization of all tested genes was done with (Ct) values of GAPDH. The conditions used for PCR amplification were: initial denaturation at 94 °C for 5 min followed by 40 cycles of 94 °C for 30 s, 58 °C for 30 s, and 72 °C for 30 sec. For mRNA expression pattern analysis, three independent experiments were performed with four replicates. Primers used for RT-PCR and qRT-PCR are listed in Table S3.

### 4.8. ROS Assay

The florescent probe 2′, 7′-dichlorodihydrofluorescein diacetate molecules (DCHDFA) from Sigma D-6883 Aldrich were used for the analysis of ROS (reactive oxygen species) production. The stock solution preparation and assay protocol was performed as previously described by [38]. In brief, day-8 BLs were collected, washed in PBS/polyvinyl pyrolidone (PVP) solution and incubated in a 10 mM/mL concentration of DCHDFA solution at 37 °C for 30 min. After a 30 min incubation, BLs were washed and visualized under a confocal laser microscope (Olympus, FV1000, Tokyo, Japan) for the florescent emission of ROS level.

### 4.9. Cell Proliferation and Apoptotic Assay

The effect of in vitro culture condition on cellular proliferation and DNA fragmentation was determined with anti-BrdU labelling and terminal deoxynucleotidyl transferase (TdT) 20-deoxyuridine, 50-triphosphate (dUTP) nick-end labeling (TUNEL) assay by using an In Situ Cell Death Detection Kit (Roche Diagnostics Corp., Indianapolis, IN, USA), as explained previously by [37,38]. Briefly, for cell proliferation assay, day-8 BLs were washed and incubated for 6 h with 100 mM BrdU at 37 °C. BLs were fixed, permeabilized, and incubated with 1 N HCL solution at room temperature (RT) for 30 min. After 1 h blocking with 3.0% BSA (bovine serum albumin), incubation with anti-BrdU (B8434-100 μL, Sigma) primary antibody followed by secondary TRITC-conjugated antibody was used to detect the BrdU labelled cells. Antibodies specifications are listed Table S4.

For the determination of apoptotic index, day-8 BLs were fixed in 4% PFA (paraformaldehyde) for 15 min. Washed and permeabilized for 30 min at room temperature in (0.5% (v/v) Triton X-100 and 0.1% (w/v) sodium citrate). Thereafter, incubated with TUNEL assay kit reagents for 1 h at 37 °C under preventive light condition. Washed and incubated with DAPI (5 min) for nuclei staining. After washing BLs were mounted on glass slides and images were captured by epifluorescence microscope (Olympus IX71, Tokyo, Japan). Image J software (National Institute of Health, Bethesda, MD, USA) were used to count the BrdU and TUNEL labelled positive nuclei from individual BL. The average percentage was determined by dividing the total cell number (stained with DAPI).

### 4.10. Nile Red Staining for Quantification of Lipid Content

A fluorescent probe (Nile red, NR) specific for detection of intracellular lipids was used to evaluate the accumulation of lipid content in the day-8 BLs. the protocol used was same as described by [36]. Briefly, fixed BLs were washed with PBS/PVP solution and incubated with 10 mg/mL NR solution for 3 h at room temperature under dark condition. Thereafter, NR stained BLs were washed with PBS/PVP solution and incubated with DAPI (5 min) for nuclei staining. The confocal laser-scanning Olympus FluoView FV1000 microscope was used to capture the images of glass slide mounted BLs. Red florescent intensities showing NR lipophilic specific probe were measured by using Image J software.

### 4.11. JC-1 Staining

A fluorochrome dye, JC-1 (Molecular probe) from (Invitrogen, Carlsbad, CA, USA) was used to determine the mitochondrial membrane potential (Ψ) in in vitro cultured BLs. The day-8 BLs were collected and fixed in 4% PFA. Washed and incubated with 10 mg/mL JC-1 dye prepared in PBS/PVP solution at 37 °C for 1 h under dark condition. In principal, the dye incorporates into mitochondria and generates either a green florescence signal by forming monomers (J-monomer) that indicates low membrane potential or a red florescence signal by forming aggregates (J-aggregate) that indicates high membrane potential. Thereafter, BLs were washed with PBS/PVP solution and stained with DAPI for 5 min. Followed by washing, BLs were mounted on a glass slide with cover slip. Images were viewed under a confocal laser scanning microscope (Olympus, FV1000, Tokyo, Japan).

### 4.12. Statistical Analysis

Statistical difference in embryonic development data were analyzed by using SPSS software version 18.0 (IBM Corp., Armonk, NY, USA). The all embryonic percentile data are presented as mean ± standard error of mean (SEM). For the analysis of imaging data, experiments were performed in triplicate sets of experiments and a single BL image was shown as representative image from the individual group. All graphical data are presented as the mean standard error of mean (SEM) curated from triplicate sets of experiments. For all the imaging data, mean fluorescence intensities were quantified per BL (*n* = 20) from individual group and histogram values were measured by using Image J software (National Institute of Health, Bethesda, MD, USA). The difference in expression level of various genes in all the groups as well as difference in the detection of fluorescence intensities were analyzed by using one-way analysis of variance followed by Sidak’s Multiple Comparison Test. GraphPad Prism 6.0 software package (USA) was used. * *p* < 0.05; ** *p* < 0.01; *** *p* < 0.001; **** *p* < 0.0001, were considered as significant differences.

## Figures and Tables

**Table 1:** Effect of BOECs condition media on embryonic development during in vitro

Groups	BOECs-CM: TCM199/SOF (Serum 2%)	Oocytes, *n*	Presumptive Zygote, *n*	Cleaved Embryos, *n* %	Total BL %	Hatched BL %
*Control*	(−)	457	430	335 (77.9 ± 0.28)	120 (28.2 ± 0.26) ^a^	44 (36.0 ± 1.87)
*BOECs-CM*	(+)	504	478	373 (78 ± 0.31)	208 (43.6 ± 0.86) ^b^	78 (37.5 ± 0.85)

^a,b^*p* > 0.05 with different superscripts indicates significant difference.

**Table 2:** Effect of different dilutions of BOECs Exosomes and BL yield during in vitro

*Groups*	BOECs-Exo Dilutions (%)	Oocytes, *n*	Presumptive Zygote, *n*	Cleaved Embryos, *n* %	Total BL %	Hatched BL %
*Control*	(−)	328	313	245 (78.4 ± 0.18)	89 (28.6 ± 0.26) ^a^	29 (32.4 ± 1.45) ^a^
*BOECs-Exo*	1	339	323	255 (78.9 ± 0.23)	120 (37.3 ± 0.49) ^c^	50 (41.8 ± 0.96) ^b,c^
	2	347	330	259 (78.6 ± 0.18)	133 (40.3 ± 0.49) ^c^	61 (45.8 ± 1.21) ^c,d^
	3	351	334	263 (78.6 ± 0.18)	151 (45.4 ± 0.68) ^d^	74 (48.9 ± 0.97) ^d^
	5	346	328	257 (78.4 ± 0.26)	105 (32.3 ± 1.32) ^b^	39 (36.8 ± 1.69) ^a,b^
	10	332	315	246 (78.4 ± 0.18)	90 (28.6 ± 0.71) ^a^	31 (33.0 ± 1.27) ^a^

^a,b,c,d ^*p* > 0.05 with different superscripts indicates significant difference.

## Supplementary Materials

Supplementary materials can be found at https://www.mdpi.com/1422-0067/21/20/7589/s1.

## Abbreviations

ART	Assisted Reproductive Technology
BOECs	Bovine Oviduct Epithelial cells
CM	Condition media
COCs	Cumulus Oocyte Complexes
Exo	Exosomes
FAO	Fatty Acid Oxidation
GLUD1	Glutamate dehydrogenase 1
IVP	In Vitro Produced
IVC	In Vitro Culture
IVF	In Vitro Fertilization
IVM	In Vitro Maturation
NTA	Nano Tracking Analysis
OXPHOS	Oxidative Phosphorylation
PDH	Pyruvate Dehydrogenase
ROS	Reactive oxygen Species
TCA-Cycle	Tri-Carboxylic Acid Cycle
