# Supplementary material for: Bovine Oviduct Epithelial Cell-Derived Culture Media and Exosomes Improve Mitochondrial Health by Restoring Metabolic Flux during Pre-Implantation Development
Source: Int J Mol Sci. 2020 Oct 14;21(20):7589. doi: 10.3390/ijms21207589 (PMC7593913; doi:10.3390/ijms21207589)
Supplement: Supplementary file 1 [file ijms-21-07589-s001.zip › Supplementary File.pdf]

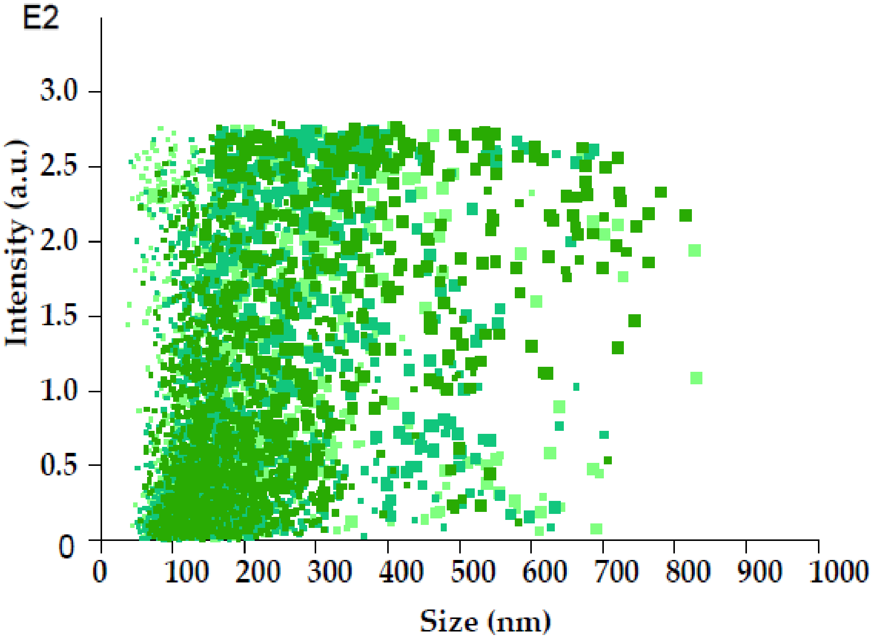

Figure 1: Nano tracking analysis showing Intensity of exosomes.

Table S1: Percentage of 8-cell stage embryo in control and BOEC-CM supplemented group.

Groups	Speculated zygote, *n*	Cleaved embryos, *n* %	8-16 cell embryos	
			n	%
**Control**	430	335 (77.9±0.28)	246	57
**BOEC-CM**	478	373 (78.1±0.31)	300	62.76

Table S2: Percentage of 8-cell stage embryo in control and BOEC-Exo supplemented group.

Groups	Speculated zygote, *n*	Cleaved embryos, *n* %	8-16 cell embryos	
			n	%
**Control**	313	245 (78.4±0.18)	181	58
**BOEC-Exo 3%**	334	263 (78.6±0.18)	227	68

Table S3: List of qRT-PCR primers.

Gene Name	Forward	Reverse	Accession #
GAPDH	TTCAACGGCACAGTCAAGG	ACATACTCAGCACCAGCATCAC	NM_001034034
CD44	CCGGAACATAGGGTTTGAGA	GGTATAACGGGTGCCATCAC	NM_174013.3
EP-CAM	ACACAGCATTCCGTCATTTG	TGGCTCAGAGCAGGAGATTT	NM_001035290.1
CD34	ATTCCCGGAAGACCTTGATT	AGTTCGGTATCAGCCACCAC	AB021662.1
CD14	TCCGTTGTGTCTGCAACTTC	TCCTCAAGCGTCAGTTCCTT	D84509.1
c-Myc	TTTTCTCCGTCCTCTGACTCTC	TTCCTCATCCTCTTGTTCTTCC	NM_001046074.2
Oct4	AGGTGTTCAGCCAAACGACTAT	GTCTCTGCCTTGCATATCTCCT	NM_174580
Esr1	ATGATGAAAGGCGGAATACG	AAGGTTGGCAGCTCTCATGT	NM_001001443.1
Ptges	GAAGAAGGCTTTTGCCAACC	TTCGGTCCGAGGAAAGAGTA	NM_174443.2
Ovgp1	GGATTTGACCACTGGTTTGG	TTTCCTTCTGGAGCAGCAGT	NM_001080216.1
INF-Ʈau	CCCCATTCTGACTGTGAAGA	TTTTGCAAGGTGGTTGATGA	AF238612.1
Ptger2	CTGCTGCTGTTGCTCATCAT	ATGGATACCCTTTCCGCTCT	NM_174588.2
ptger4	TCGCTTAGGACTCTGCGAAT	TCACTGGGAAACGTGACTTG	NM_174589.2
Plac8	TGAGGACCCTCTACAGGACTCG	TAGAAAGTGCGATTGGCTCTCC	XM_024993443.1
PAG1	GAACGAAATTAAGGAGGTGACG	AGCAAAGTCTGTTCTGCTGTTG	XM_005215558.4
IGF-1R	GATCCCGTGTTCTTCTACGTTC	CAGCCTGCTGCTATTTCTTTTT	NM_001244612.1
MnSOD2	CGCTGGAGAAGGGTGATGTTAC	GTTTGATGGCTTCCAGCAATTC	NM_201527.2
GSR	CCTATGTGAGCCGCCTGAAC	GGCGATCAGGATGTGAGGAG	NM_001114190.2
GPX4	GCACGAATTTTCAGCCAAGG	AAACCACACTCGGCGTATCG	NM_001346431.1
Bcl2	TGACTTCTCTCGGCGCTACC	CACATGACCCCTCCGAACTC	XM_024984176.1
Bax	GTGCCCGAGTTGATCAGGAC	AGGGACAGCAGGCACTTCAG	XM_015458140.2
PPARα	ATATTTCCCTCTTTGTGGCTGC	ATGGTTGTTCTGTAGGTGGAGT	XM_024991367
Cpt1a	GAGGGAGACTTTACACGGATGA	AGATGTATTCCTCCCACCAGTC	NM_001304989
Pdk4	GGTGATTGTTGTCTTGGGGAAA	AATTATCCATCACAGGCGTTGG	NM_001101883

Table S4: List of Antibodies used.

Target	Cat #	Manufacturer
Anti-Brdu	B-8434	Sigma
DAPI	62248	Thermo Fisher scientific
TRITC	A6071	Invitrogen
